# Bias Against Older Adults in Young Children and Their Parents

**DOI:** 10.1093/geroni/igab046.3568

**Published:** 2021-12-17

**Authors:** Jenny Jaquet (née Bauer), Jennifer Bellingtier

**Affiliations:** Friedrich Schiller University Jena, Jena, Thuringen, Germany

## Abstract

Parents are an important source of social learning for their children. However, little is known about whether they play a role in shaping ageist attitudes in children. We investigated how parents’ biases against older adults would relate to those of their children and how preferences would differ depending on the child’s age. Participants were 56 parent-child dyads with the children’s age ranging from four to eight years (parents mean age = 36.95, SD = 5.49). Children were divided into three age groups, preschool (n = 18), early school-aged (n = 18), and middle school-aged (n = 20). Children and parents completed a picture rating task, which included the evaluation of 28 images of younger and older adults faces. Children used a smiley-face rating scale on a touch-screen computer, and parents used a sliding preference scale for their ratings. It was found that both, children (t(55) = 5.47, p < .001, d = 0.73) and their parents (t(55) = 2.05, p = 0.045, d = 0.27), gave significantly more positive ratings to younger than to older adults, which is consistent with an underlying bias for younger adults. Contrary to our expectations, this preference in children held across age groups and was not associated with parental preferences. Nevertheless, it has been shown that ageist preferences can already be detected in childhood. Further longitudinal research is needed to track the development of ageism from childhood on, and efforts to combat ageism should be addressed not only to adults, but to children as well.

